# Repeat-mediated genetic and epigenetic changes at the *FMR1* locus in the Fragile X-related disorders

**DOI:** 10.3389/fgene.2014.00226

**Published:** 2014-07-17

**Authors:** Karen Usdin, Bruce E. Hayward, Daman Kumari, Rachel A. Lokanga, Nicholas Sciascia, Xiao-Nan Zhao

**Affiliations:** Section on Gene Structure and Disease, Laboratory of Cell and Molecular Biology, National Institute of Diabetes, Digestive and Kidney Diseases, National Institutes of Health, BethesdaMD, USA

**Keywords:** Fragile X-related disorders, FX-associated tremor/ataxia syndrome (FXTAS), FX-associated primary ovarian insufficiency (FXPOI), Fragile X syndrome (FXS), repeat expansion disease

## Abstract

The Fragile X-related disorders are a group of genetic conditions that include the neurodegenerative disorder, Fragile X-associated tremor/ataxia syndrome (FXTAS), the fertility disorder, Fragile X-associated primary ovarian insufficiency (FXPOI) and the intellectual disability, Fragile X syndrome (FXS). The pathology in all these diseases is related to the number of CGG/CCG-repeats in the 5′ UTR of the Fragile X mental retardation 1 (*FMR1*) gene. The repeats are prone to continuous expansion and the increase in repeat number has paradoxical effects on gene expression increasing transcription on mid-sized alleles and decreasing it on longer ones. In some cases the repeats can simultaneously both increase *FMR1* mRNA production and decrease the levels of the *FMR1* gene product, Fragile X mental retardation 1 protein (FMRP). Since FXTAS and FXPOI result from the deleterious consequences of the expression of elevated levels of *FMR1* mRNA and FXS is caused by an FMRP deficiency, the clinical picture is turning out to be more complex than once appreciated. Added complications result from the fact that increasing repeat numbers make the alleles somatically unstable. Thus many individuals have a complex mixture of different sized alleles in different cells. Furthermore, it has become apparent that the eponymous fragile site, once thought to be no more than a useful diagnostic criterion, may have clinical consequences for females who inherit chromosomes that express this site. This review will cover what is currently known about the mechanisms responsible for repeat instability, for the repeat-mediated epigenetic changes that affect expression of the *FMR1* gene, and for chromosome fragility. It will also touch on what current and future options are for ameliorating some of these effects.

## INTRODUCTION

The Fragile X-related disorders (FXDs) are members of a large and growing group of human genetic conditions known as the repeat expansion diseases (Fry and Usdin, 2006). These diseases all have an unusual mutational mechanism: the causative lesion is an increase in the number of repeats (“expansion”) at a specific tandem repeat tract. In the case of the FXDs, the repeat unit is CGG/CCG and the repeat tract is located on the long arm of the X chromosome in the 5′ untranslated region of the Fragile X mental retardation 1 (*FMR1*) gene (Fu et al., 1991; Verkerk et al., 1991). This gene encodes Fragile X mental retardation 1 protein (FMRP), a protein important for learning and memory. Increasing repeat number is associated with increased instability of the repeat tract (Ashley-Koch et al., 1998; Nolin et al., 2011). The repeat number also has bearing on the severity of the FXD symptoms although, as will be described in more detail later, the relationship between repeat number and pathology is not always linear.

Alleles with <45 repeats are considered to be clinically unaffected and to have a very low risk of expansion, while alleles with >54 repeats confer risk of one or more of the FXDs as well as some risk of further expansion. Two pathological allele size classes are usually distinguished: alleles with 55–200 repeats are considered to be premutation (PM) alleles, while alleles with >200 repeats are referred to as full mutation (FM) alleles. PM alleles confer risk of an adult-onset neurodegenerative disorder known as Fragile X-associated tremor/ataxia syndrome (FXTAS) and/or a form of ovarian dysfunction known as Fragile X-associated primary ovarian insufficiency (FXPOI). In contrast, FM alleles are associated with Fragile X syndrome (FXS), the leading heritable cause of intellectual disability (Fu et al., 1991; Verkerk et al., 1991).

This review will briefly summarize some of the clinical features of the FXDs (the reader is referred to much more comprehensive discussions of this topic elsewhere in this issue) and then discuss current thinking about the underlying expansion mutation responsible for these diseases along with two specific consequences of this expansion, the chromosome fragility that gives these disorders their name and the repeat-mediated epigenetic changes that contribute to disease pathology.

### FRAGILE X-ASSOCIATED TREMOR/ATAXIA SYNDROME

Fragile X-associated tremor/ataxia syndrome is an adult onset neurodegenerative disorder whose symptoms include cerebellar ataxia and intention tremor [reviewed in (Hagerman, 2013)]. Cognitive decline or impairment, peripheral neuropathy, Parkinsonism, and urinary and bowel incontinence may also be seen. Magnetic resonance imaging (MRI) findings include global brain atrophy, enlarged ventricles, white matter disease, and increased signals in the middle cerebellar peduncle. In addition, characteristic intranuclear inclusions are seen in the brains and other organs of affected individuals. These inclusions are tau- and synuclein negative but contain *FMR1* mRNA (Tassone et al., 2004) and a wide variety of other proteins (Iwahashi et al., 2006; Sellier et al., 2013). In general the severity of the FXTAS symptoms are directly related to the length of the CGG/CCG -repeat tract (Leehey et al., 2008) and there is a similar relationship between repeat number and the extent of *FMR1* transcription in the PM range (Kenneson et al., 2001). However, the clinical symptoms and rate of progression of FXTAS vary and a life expectancy of anywhere between 5 and 25 years after the onset of symptoms is seen (Seritan et al., 2008). Males tend to be more severely affected than females, due at least in part to the protective effect of the second X chromosome in females (Berry-Kravis et al., 2005; Jacquemont, 2005; Coffey et al., 2008; Rodriguez-Revenga et al., 2009; Tassone and Hagerman, 2012).

### FRAGILE X PRIMARY OVARIAN INSUFFICIENCY

Fragile X-associated primary ovarian insufficiency is an ovarian dysfunction disorder that presents with a spectrum of involvement ranging from heavy bleeding, irregular periods or increased rates of twinning, to infertility and menopause before the age of 40 (Sherman, 2000; Allen et al., 2007; Wittenberger et al., 2007). FXPOI is seen in ∼20% of females carrying a PM allele (Sullivan et al., 2011) and the PM is the leading cause of early menopause in the general population (Murray et al., 2014). Even women who carry the PM but do not meet the strict definition of FXPOI, tend to reach menopause on average 5 years earlier than their sisters without the PM (Murray et al., 2000, 2014; Sullivan et al., 2005). Women with FXPOI show signs of early ovarian aging including shorter than normal menstrual cycles than women who are still cycling, increased twinning, reduced levels of anti-müllerian hormone (AMH) indicating a reduced follicle pool, and elevated follicle stimulating hormone (FSH; Welt et al., 2004; Rohr et al., 2008). The risk of FXPOI shows an unusual U-shaped relationship with repeat number with the highest risk being associated with alleles that have 80–99 repeats (Ennis et al., 2006; Allen et al., 2007; Tejada et al., 2008).

### FRAGILE X SYNDROME

Fragile x syndrome is the most common heritable cause of intellectual disability and the most common known monogenic cause of autism [reviewed in (McLennan et al., 2011)]. It is associated with a wide range of symptoms of varying severity that may include speech and language delay, macroorchidism in males, hyperarousal and depression. Most males and 25% of females have cognitive impairment (IQ < 70), while nearly all patients present with behavior problems, males typically with attention deficit hyperactivity disorder (ADHD) and aggression and females with shyness and social withdrawal (Hagerman and Hagerman, 2008). As many as 67% of male FM carriers meet the criteria for autism or autism spectrum disorder (ASD; Clifford et al., 2007; Wang et al., 2010). Seizures are seen in 10–20% of affected children (Berry-Kravis, 2002). In ∼10% of children there is also a so-called Prader–Willi phenotype that includes severe obesity, hyperphagia and hypogonadism or delayed puberty (Nowicki et al., 2007). At the cellular level, FXS is associated with immature dendritic spine morphology (Braun and Segal, 2000; Irwin et al., 2000). Amongst the effects seen at the molecular level there is dysregulated protein synthesis in the postsynaptic density in response to activation of the mGluR5 receptor that results in a net increased excitability of neuronal circuits (Bear et al., 2004; Dolen and Bear, 2008).

The FM allele is also associated with the expression of a folate-sensitive fragile site for which the FXDs are named. This site appears as a gap, constriction or break that is seen in metaphase chromosomes when cells are subjected to folate-stress. In addition to this chromosomal abnormality, a number of female FM carriers have been shown to be mosaic for Turner syndrome (TS), a disorder in which one of the two X chromosomes has been lost (Shapiro et al., 1994; Tejada et al., 1994; Wilkin et al., 2000). The symptoms of TS include short stature, scoliosis, gonadal dysfunction, and cognitive problems including difficulties with spatial-temporal processing. Analysis of fetuses with the FM suggests that the risk of TS is significantly higher in female FM carriers than it is in the general population (Dobkin et al., 2009). Furthermore, unlike TS in the general population where the paternal X chromosome is more likely to be lost (Pelotti et al., 2003), in FX-related TS, it is the maternally transmitted chromosome carrying the FM allele (Dobkin et al., 2009).

### FXS IS A LOSS-OF-FUNCTION DISORDER WHILE FXTAS AND FXPOI ARE GAIN-OF-FUNCTION DISORDERS

Individuals carrying intragenic loss of function mutations in the *FMR1* gene show symptoms very similar to those carrying repeat expansions in the FM range (De Boulle et al., 1993; Gu et al., 1994; Lugenbeel et al., 1995; Myrick et al., 2014) and the disruption of the *FMR1* gene in mice leads to the recapitulation of some aspects of FXS pathology including the increased density of immature dendritic spines (Comery et al., 1997). These data support the idea that FXS results from a failure to produce functional FMRP (Pieretti et al., 1991; Sutcliffe et al., 1992).

Since individuals with FXTAS and FXPOI make more FMRP than FM carriers who do not show signs of neurodegeneration or ovarian dysfunction, the symptoms of these disorders are thought to result from a gain of function of the transcript containing a large CGG-repeat tract. This gain of function may be related to the ability of transcripts containing the repeats to sequester CGG-repeat binding proteins, such as Sam68 (Sellier et al., 2010), DROSHA and DCR8 (Sellier et al., 2013) and Pur-alpha (Jin et al., 2007) and thus prevent their normal function, or to the fact that the repeats facilitate the generation or stabilization of a toxic protein that results from translation of the *FMR1* transcript that is initiated at non-ATG codons (Todd et al., 2013).

### THE CLINICAL PRESENTATION OF THE FXDs CAN BE VERY VARIABLE

A wide range of symptoms is seen in all three disorders and both FXTAS and FXPOI are incompletely penetrant (Jacquemont et al., 2004; Sullivan et al., 2005; Ennis et al., 2006; Allen et al., 2007; Tejada et al., 2008; Hagerman, 2013). In the case of FM carriers, a number of individuals do not meet the criteria for FXS having only mild symptoms reminiscent of FXS and an IQ within or close to the normal range (Hagerman et al., 1994; Wohrle et al., 1998; Taylor et al., 1999; Tassone et al., 2000b; Loesch et al., 2004, 2012; Tabolacci et al., 2008a; Santa Maria et al., 2013).

Furthermore, it is becoming apparent that there is some overlap in the clinical symptoms in PM and FM carriers. For example, small intranuclear inclusions characteristic of FXTAS have been reported in males with FM alleles who still make some *FMR1* mRNA and some FM carriers have symptoms and MRI findings characteristic of FXTAS (Loesch et al., 2012; Santa Maria et al., 2013). Conversely, symptoms of ADHD, seizures, shyness, social deficits, ASD and occasionally intellectual disability that are more typical of FXS are also sometimes seen in PM carriers (Hagerman et al., 1996; Farzin et al., 2006; Grigsby et al., 2006, 2007; Hessl et al., 2007; Chonchaiya et al., 2012; Kim et al., 2014). In addition, increased *FMR1* transcription and/or FXTAS symptoms have also been in reported in some carriers of *FMR1* alleles that have 45–54 repeats, so-called gray zone alleles (Kenneson et al., 2001; Hall et al., 2011, 2012; Liu et al., 2013). These alleles have also been suggested to contribute to the etiology of disorders associated with parkinsonism (Loesch et al., 2007, 2009; Hall et al., 2011; Trost et al., 2013). Some reports have also linked these alleles to ovarian dysfunction (Bretherick et al., 2005; Bodega et al., 2006; Streuli et al., 2009; Ishizuka et al., 2011; Karimov et al., 2011; Pastore et al., 2012; Barasoain et al., 2013) although others have found no such association (Bennett et al., 2010; Voorhuis et al., 2013; Murray et al., 2014).

## REPEAT INSTABILITY AT THE FX LOCUS

The PM repeat tract is at risk of expansion on intergenerational transmission in humans. There is also some evidence of expansions occurring in some somatic tissues including the brain (Lokanga et al., 2013). Both small and large expansions are seen. Small expansions, which are seen more frequently on paternal transmission, give rise to larger PM alleles that affect the risk of FXTAS and FXPOI. The resultant larger alleles are also at increased risk of further expansions (Heitz et al., 1992). Large expansions give rise to FM alleles and these are exclusively maternally transmitted (Rousseau et al., 1991; Nolin et al., 1996; Ashley-Koch et al., 1998). In the gametes of male fetuses with FMs only FM alleles are observed (Malter et al., 1997), yet post-natally most FM males only have PM-sized alleles in their sperm (Reyniers et al., 1993; Rousseau et al., 1994). This would be consistent with the idea that expansions can occur in both males and females but that there is selection against large expansions during spermatogenesis. While expansions predominate, contractions are also seen including reversions of PM alleles into the normal size range (Mornet et al., 1996; Vaisanen et al., 1996; Gasteiger et al., 2003; Tabolacci et al., 2008b) and FMs into the PM range (Malzac et al., 1996; Loesch et al., 1997). It is some combination of these expansions and contractions that accounts for the repeat length mosaicism that is often seen in PM and FM carriers (Rousseau et al., 1991; Nolin et al., 1994; Prior et al., 1995; Cohen et al., 1996; de Graaff et al., 1996; Dobkin et al., 1996; Maddalena et al., 1996; Mila et al., 1996; Grasso et al., 1999; Petek et al., 1999; Schmucker and Seidel, 1999; Garcia Arocena et al., 2000; Fan et al., 2005; Govaerts et al., 2007; Todorov et al., 2009; Ferreira et al., 2013; Pretto et al., 2013; Santa Maria et al., 2013).

The mechanism of repeat instability is not fully understood. Both strands of the FX repeats are able to form a variety of intrastrand folded structures including hairpins and quadruplexes containing a mixture of Watson–Crick and Hoogsteen base interactions (Fry and Loeb, 1994; Mitas et al., 1995; Nadel et al., 1995; Usdin and Woodford, 1995; Usdin, 1998; Fojtik and Vorlickova, 2001; Renciuk et al., 2009). The ability to form such structures is a common feature of those diseases arising from repeat expansion (reviewed in Usdin, 2008). This has led to the suggestion that expansion arises from a problem related to the formation of these structures. In addition to gender, two key factors have been identified that affect expansion risk in humans. These are the length of the repeat tract and the presence or absence of AGG-interruptions within the tract (Eichler et al., 1994; Zhong et al., 1996; Kunst et al., 1997; Nolin et al., 2003, 2011, 2013; Yrigollen et al., 2012). *In vitro* studies show that the presence of an AGG-repeat interruption diminishes the stability of the secondary structures formed by a CGG-repeat tract (Weisman-Shomer et al., 2000; Jarem et al., 2010). Thus the fact that AGG-interruptions reduce instability would be consistent with a role of these structures in repeat expansion.

Repeat instability has been studied in a number of mouse models of the PM (Bontekoe et al., 1997; Lavedan et al., 1998; Peier and Nelson, 2002; Fleming et al., 2003; Entezam et al., 2007; Brouwer et al., 2008). While small expansions are seen at a high frequency in mouse models with ∼130 repeats, the large expansions characteristic of the generation of a FM allele in humans are only rarely seen in these animals. However, longitudinal studies of the size of the transmitted allele in mice also demonstrates that alleles can undergo multiple rounds of expansion over time, with the changes in the repeat number in the transmitted allele increasing with increasing repeat number (Lokanga et al., 2013; Zhao and Usdin, 2014). This raises the possibility that the large expansions seen in humans may arise from the accumulated effect of a series of small expansions. Since the perigametic interval, the time between the last premeiotic cell division in the gamete of the parent and the first mitotic division in the offspring, usually lasts two decades or more in humans compared to a few weeks or months in rodents, humans have a much larger window of opportunity during which repeat units can be added. The incremental accumulation of additional repeats with time may also account for the observed parental age effect on the risk of expansion seen in humans (Ashley-Koch et al., 1998). In addition, PM alleles with 190 repeats show much larger expansions than PM alleles that only have 130 repeats, raising the possibility that the threshold for large expansions is higher in mice than it is in humans (Entezam et al., 2007).

### MODELS FOR REPEAT EXPANSION

**FIGURE 1:**
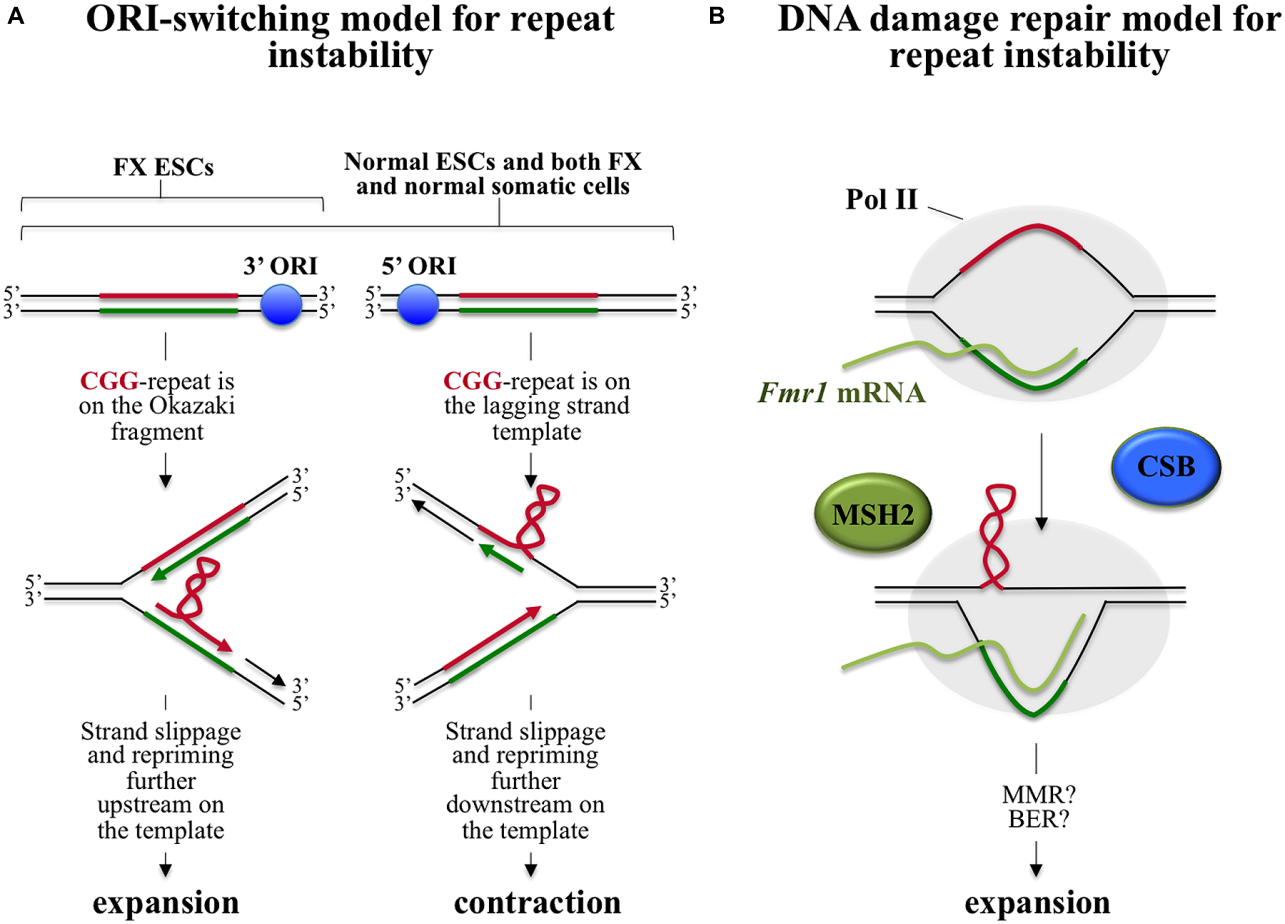
**Models for intergenerational repeat instability at the FX locus.** In both models the CGG-rich strand, which forms the most stable secondary structure, is shown in red and the CCG-rich strand is shown in green. **(A)** ORI-switch model for FX repeat instability. The *FMR1* gene is flanked by two ORIs, one located 45 kb upstream (5′) and another 45 kb downstream (3′) of the gene (Gerhardt et al., 2014). During replication transient dissociation of the Watson and Crick strands of DNA can occur with slippage of the two strands relative to one another. Priming of DNA synthesis can then occur from the slipped position. This strand-slippage process is thought to occur more commonly during lagging strand replication and is seen more frequently when the template or nascent strand have the potential to form secondary structures. Secondary structure formation by the lagging strand template would result in loss of repeats/contractions since repriming 5′ of the structure on the template would be favored, while the formation of such structures on the corresponding Okazaki fragment would favor addition of repeats since repriming would then be more likely to occur more 3′ on the template. Replication from the upstream ORI results in the CGG-rich strand being on the lagging strand template (note that only one side of the bidirectional replication fork is shown). In contrast, replication from the downstream ORI results in the CGG-rich strand being on the Okazaki fragment. In somatic cells replication proceeds equally well from both ORIs resulting in no net gain of repeats. However, in FX ESCs it is suggested that replication predominantly occurs from the downstream ORI resulting in a net gain of repeats. **(B)** A DNA repair based model for FX repeat instability. During transcription, RNA Polymerase II (Pol II) occludes the template strand, leaving the non-template strand free to form secondary structures. Secondary structure formation may also be facilitated by the co-transcriptional formation of stable RNA:DNA hybrid in the repeat region (Loomis et al., 2014). These structures are then processed via an MSH2-dependent expansion pathway in which CSB plays a supportive role. This pathway is initiated by binding of the MSH2-containing complex to the mismatches in the structures. Events downstream of this binding are not currently known. One possibility is that classical mismatch repair is initiated in response to MSH2 binding but that hairpins form in the flaps generated by strand displacement synthesis as part of this repair. Hairpin formation may prevent normal removal of these bases by flap endonucleases (Spiro et al., 1999) resulting in their incorporation into the “repaired” strand.

There are a number of different models for repeat expansion, some of which are based on the idea that expansion results from some problem associated with DNA synthesis through the repeats during normal genomic replication and others that suggest that expansion occurs as a result of aberrant repair of the secondary structures formed by the repeats perhaps during transcription or as a result of DNA damage (see Pearson et al., 2005; Mirkin, 2007 for comprehensive reviews). One of these models, the ORI-switch model, proposes that expansion occurs during normal genome replication as the result of a switch or change in the origin of replication (ORI) used to replicate the locus in question. This switch results in a change in the direction of replication through the repeat (Mirkin and Smirnova, 2002). This switch could potentially occur in the embryo when cell division is very rapid and additional ORIs are needed to complete replication timeously. This model is predicated on the premise that expansions and contractions occur via strand-slippage during replication, a process more likely to occur during lagging strand DNA synthesis. The most stable secondary structure is thought to be the one most likely to promote slippage since it would favor repriming of DNA synthesis from the slipped position. Since the secondary structures formed by the CGG-repeats are more stable than those formed by the CCG-repeats, this model would predict that replication from an upstream ORI, that results in the CGG-repeats being on the lagging strand template, would favor contractions since repriming by the nascent strand after strand-slippage would likely occur 5′ of the structure on the template as illustrated in **Figure 1A**. In contrast, replication from a downstream ORI would result in the CGG-repeats being on the nascent Okazaki fragment and would thus favor expansions since repriming by the nascent strand further 3′ on the template would occur more often.

In normal human embryonic stem cells (ESCs) and in differentiated cells with either normal or FX alleles, replication proceeds equally well from ORIs located >45 kb upstream and downstream of the repeat (Gerhardt et al., 2014). However, in FX ESCs replication proceeds predominantly from the downstream ORI such that the CGG-strand would be on the Okazaki fragment consistent with the requirements of the ORI-switch model (Gerhardt et al., 2014). Since in somatic cells replication through the *FMR1* locus proceeds from both origins, the prediction of the model would be that both expansions and contractions can occur with equal probability resulting in no net gain of repeats. In contrast, since in FX ESCs replication proceeds predominantly from the downstream ORI, expansions would predominate in these cells. However, since the change in ORI usage seen in FX ESCs does not occur in normal ESCs, the switch in ORI site usage is not an intrinsic feature of the *FMR1* locus in this cell type. This raises the question of what causes the ORI-switch in FX cells in the first place. Further work is needed to understand whether the change in ORI usage in FX ESCs is a consequence of the expansion event that gave rise to the FX allele or the cause. Since the *FMR1* gene is already methylated in the FX ESCs studied, it would be useful to examine ESCs from individuals with unmethylated FM alleles to assess the role of DNA methylation in ORI switching.

While it is possible that problems at the replication fork are responsible for expansion, there are a number of lines of evidence that support other types of models. For example, expansion is known to be high in tissues with a low proliferative capacity like brain and liver and in a mouse model of the FX PM expansion is seen in post-mitotic cells such as oocytes and neurons (Lokanga et al., 2013). This, along with data emerging from other repeat expansion diseases, suggests that genomic replication may not be required for expansion (Lia et al., 1998; Fortune et al., 2000; Ishiguro et al., 2001; Kovtun and McMurray, 2001; Lokanga et al., 2013, 2014b). Furthermore, oxidative damage has been shown to be risk factor for expansion in a PM mouse model (Entezam et al., 2010), supporting the idea that an aberrant DNA damage response rather than a problem with replication may be responsible for expansion. This idea is bolstered by the finding that mutations in the genes for OGG1 and NEIL1, two DNA glycosylases involved in base excision repair (BER) of oxidized bases, decrease the expansion frequency in a mouse model of Huntington Disease, a Repeat Expansion Disorder involving CAG-repeats (Kovtun et al., 2007; Mollersen et al., 2012).

The mismatch repair (MMR) protein MSH2 has been shown to be essential for both intergenerational and germ line expansions in the FX PM mouse (Lokanga et al., 2014b). MSH2 has also been implicated in expansion in mouse models of other Repeat Expansion Diseases (Manley et al., 1999; Savouret et al., 2003). In addition to providing clues as to the mechanism of expansion, the effect of parental *Msh2*-heterogosity in the FX PM mouse also tells us something about the timing of these expansions. Specifically, the *Msh2*^-/-^ offspring of *Msh2*^+/-^ parents have the same expansion frequency as their *Msh2*^+/+^ and *Msh2*^+/-^ littermates. Thus, expansion events detected at birth are predominantly the result of expansion events occurring prezygotically. This may be pertinent to the question of whether expansions seen on intergenerational transmission in humans occur prezygotically or postzygotically (Moutou et al., 1997; Reyniers et al., 1999; Huang et al., 2014).

MSH2 likely binds the FX DNA hairpins as part of the MuxtSβ complex, as reported for the hairpins formed by CAG/CTG-repeats (Owen et al., 2005; Lang et al., 2011). MutSβ, a heterodimer of MSH2 and MSH3, normally binds and triggers the repair of insertion-deletion (IDL) loops of 1–15 nucleotides. The FX repeats form hairpins with G•G and C•;C mismatches (Mitas et al., 1995; Usdin and Woodford, 1995; Yu et al., 1997; Usdin, 1998) and it is likely that binding to the FX repeats occurs via the recognition of these mismatched bases. However, whether MSH2 is acting via classical MMR to recruit other MMR proteins or via another DNA repair pathway, like BER, in which MSH2 also participates, is currently unclear.

While expansion in the mouse model does not seem to require genomic replication, it does require transcriptionally competent chromatin since expansion only occurs when the PM allele is situated on the active X chromosome (Lokanga et al., 2014a) This is consistent with observations from humans, that methylated alleles that are transcriptionally inactive, are stable (Wohrle et al., 2001). Loss of Cockayne syndrome B (CSB), a protein essential for transcription coupled repair (TCR), a DNA repair pathway that is unique to actively transcribed genes, affects germ line, and somatic expansion risk in the PM mouse (Zhao and Usdin, 2014). It also causes a decrease in the extent of somatic expansion in some organs but not others indicating that CSB facilitates, but is not essential for expansion. Since TCR requires CSB, its non-essential role suggests that the expansion process does not involve TCR itself. It may be that CSB is acting via its ability to facilitate steps in the BER pathway (Muftuoglu et al., 2009; Menoni et al., 2012; Aamann et al., 2013).

Since TCR is not essential for somatic expansions, the fact that expansion only occurs on the active X chromosome must have a different molecular basis. It may simply be that occlusion of the template strand by the RNA polymerase II (Pol II) during transcription increases the opportunity for the formation of the secondary structures on the non-template strand that act as the substrates for expansion. Alternatively, the effect of transcription may be mediated via the formation of persistent RNA:DNA hybrids or R-loops at the *FMR1* locus (Loomis et al., 2014). These R-loops may allow the expansion substrates to form because reannealing of the duplex behind Pol II cannot take place. These data suggest a model for repeat instability in which transcription results in the formation of the secondary structures that act as the substrate for the MSH2-dependent, CSB-facilitated expansion pathway as illustrated in **Figure 1B**.

### POTENTIAL APPROACHES TO REDUCING EXPANSION RISK

While repeat length and purity seem to be the most important factors driving expansion of PM alleles in humans, it may be that other genetic or environmental factors contribute to expansion risk in smaller PM alleles or in gray zone/intermediate alleles. The fact that ATM protects the genome against repeat expansion in mice (Entezam and Usdin, 2008, 2009) is of interest in this regard since ATM mutations are relatively common in the human population (Swift et al., 1986) and may thus be a relatively common source of variability in the extent of expansion in carriers of smaller alleles. Our demonstration that oxidative stress increases expansion risk in mice is of general interest since there are many sources of oxidative stress to which both rodents and humans are exposed. These include internal sources, resulting from normal metabolism, and external sources in the form of environmental pollutants, ionizing and ultraviolet radiation, heat shock, and sources of inflammation. However, whether dietary antioxidants can protect against expansions is not known.

The contribution of the MSH2 binding partners MSH3 and MSH6 to repeat expansion in FX or the FX PM mice is not known. However, while MSH6 has been shown to be important for expansion in Friedreich ataxia (FRDA) induced pluripotent stem cells (Du et al., 2012), in mouse models of other repeat expansion diseases, it is MSH3 that is important (van den Broek et al., 2002; Foiry et al., 2006; Tome et al., 2013). It has been suggested that the development of MSH3 inhibitors may have therapeutic potential since loss of MSH3 is less deleterious than the loss of either MSH2 or MSH6 (Foiry et al., 2006; Halabi et al., 2012). Furthermore, the data suggest that improving the efficacy of the pathways that lead to contractions or error-free repair could in principle reduce expansion risk.

## REPEAT-MEDIATED EPIGENETIC EFFECTS

Curiously, CGG-repeats can both enhance and repress *FMR1* gene expression, causing hyperexpression of PM alleles and hypoexpression or silencing of FM alleles. The mechanisms involved are not at all well understood.

### HYPEREXPRESSION OF THE PM ALLELE

Fragile X mental retardation 1 mRNA is expressed at elevated levels in cells of humans (Tassone et al., 2000c) and mice with the PM allele (Entezam et al., 2007; Brouwer et al., 2008). These levels are directly related to repeat number and can be as high as 10 times that of the *FMR1* transcript in normal cells. Since the pathology seen in PM carriers is thought to be related either to the ability of the RNA to sequester proteins or the toxic proteins that can be made from the PM transcripts, the elevated levels of PM mRNA is likely to contribute to disease severity. The increased RNA levels are the result of increased transcription initiation rather than increased transcript stability (Tassone et al., 2007). Thus the CGG-repeats may act as a downstream enhancer/modulator of transcription. The increase in mRNA is also correlated with changes in transcription start site usage in which larger PM alleles initiate transcription from upstream start sites more frequently than occurs in normal cells (Beilina et al., 2004). This is suggestive of an altered chromatin conformation on the PM allele.

The increase in *FMR1* transcription in PM carriers is known to be associated with an increased abundance of acetylated histones at the *FMR1* promoter (Todd et al., 2010), however, whether this is a cause or consequence of the increased transcription remains to be determined. Long tracts of CGG-repeats have been shown to exclude nucleosomes *in vitro* (Wang et al., 1996). Should this also occur *in vivo* it could potentially lead to increased transcription by increasing the accessibility of transcription factors to the promoter. The R-loops formed by the CGG-Repeats (Groh et al., 2014; Loomis et al., 2014) could also play a role in *FMR1* hyperexpression. R-loops may be less prone to assemble nucleosomes (Dunn and Griffith, 1980) and more prone to chromatin decondensation (Powell et al., 2013). The effect of the longer R-loops formed on an expanded repeat may extend further into the flanking regions perhaps enhancing binding of the promoter by transcription factors or chromatin modifiers that in turn promote transcription initiation. It is also possible that the FX repeats directly bind factors that can remodel chromatin or regulate *FMR1* transcription. For example, pur alpha and pur beta are multifunctional proteins that can bind CGG-repeats very effectively and in some contexts are known to activate transcription (Huang et al., 2009).

### REPEAT-MEDIATED GENE SILENCING OF FM ALLELES

Most FM alleles are largely or completely silenced. How this silencing is accomplished is not well understood or how it is that some carriers of FM alleles escape this silencing. The 5′ end of the *FMR1* gene in FXS-derived patient cell lines is hypermethylated and associated with hypoacetylated histones (Chiurazzi et al., 1998, 1999; Coffee et al., 1999; Pietrobono et al., 2002, 2005; Biacsi et al., 2008). In addition, histone H3 is hypomethylated on lysine 4 (H3K4) on FX alleles and enriched for dimethylated H3K9 and trimethylated H3K27, marks typical of developmentally regulated genes, as well as trimethylated H3K9 and trimethylated H4K20, marks typically seen on constitutive heterochromatin like the tandem repeats that make up the pericentric heterochromatin (Kumari and Usdin, 2010). However, when these modifications are deposited and the sequence of events involved is unclear.

A study of FMRP expression in chorionic villi (CV) of two male Fragile X fetuses led to the suggestion that silencing of the FX allele occurs in the CV between 10 and 12.5 weeks of age (Willemsen et al., 2002). However, in other studies DNA methylation of the FX allele was already detectable in CV samples of 8–10 week old fetuses (Devys et al., 1992). In general, reprogramming of DNA methylation is thought to occur earlier in the embryo than in trophectoderm-derived cell lineages (Santos et al., 2002). Whether FX gene methylation also occurs earlier in the embryo remains to be seen but would be consistent with the observation that 2 out of the 3 ESC lines that have been derived so far are already methylated (Colak et al., 2014; Gerhardt et al., 2014).

In a study of an unmethylated FX ESC line, teratomas derived from these ESCs showed a ∼20-fold reduction in transcription along with enrichment for H3K9 methylation, while the DNA was still unmethylated (Eiges et al., 2007). This suggests DNA methylation is a relatively late event in the silencing process. Other evidence that supports this idea is the fact that carriers of unmethylated FM alleles (UFMs) show evidence of H3K9 dimethylation characteristic of silenced alleles but no DNA or H3K27 methylation (Tabolacci et al., 2008a). Furthermore, treatment of patient cells with the demethylating agent 5-azadeoxycytidine (AZA) reduces DNA methylation but does not affect the levels of H3K9 methylation (Chiurazzi et al., 1998, 1999; Coffee et al., 1999; Pietrobono et al., 2002, 2005; Biacsi et al., 2008). The idea that DNA methylation is a relatively late event in the silencing process would be consistent with the observation that *de novo* methylation of many other genes is associated with prior histone methylation (Feldman et al., 2006; Vire et al., 2006; Dong et al., 2008; Epsztejn-Litman et al., 2008; Tachibana et al., 2008).

A contrasting picture of events emerges from a study of two other ESC lines. In these cells H3K9 dimethylation and loss of *FMR1* transcription was only seen after >45 days of neuronal differentiation (Colak et al., 2014). However, the FM allele in these ESCs was already at least partially methylated as evidenced by the resistance of the promoter to *Eag* I digestion. Thus presumably early events in the silencing process had already taken place prior to neuronal differentiation. Nonetheless, these data suggest that in this system H3K9 dimethylation occurs many weeks after DNA methylation had begun, an observation that is perplexing given the normally close linkage between these events.

A key unresolved question in the field is what is the trigger for gene silencing. The distribution of histone modifications on the FX allele may provide some insight into where the target may be. For example, H3K9me3 and H4K20me3, marks typical of constitutive heterochromatin, show a peak of enrichment in the region of the repeat, while the other histone modifications are more uniformly distributed across the 5′ end of the gene. The enrichment of H3K9me3 and H4K20me3 would be consistent with the idea that the repeats themselves are the early target of the silencing process. Deposition of other histone marks more typical of developmentally regulated genes onto the *FMR1* 5′ end may be a consequence of the loss of a proposed boundary element located between the *FMR1* gene and the zone of heterochromatin found upstream of the *FMR1* gene on normal and affected alleles (Kumari and Usdin, 2010). The nature of the boundary element is unclear but while CCCTC-binding factor (CTCF) binds to this region, this factor is unlikely to be involved (Lanni et al., 2013). It is also possible that the broader distribution of H3K9me2 and H3K27me3 reflects a greater ability of these heterochromatin marks to spread than the marks of constitutive heterochromatin. In either event, the data support the idea that the initiation of silencing begins in the repeat itself (Kumari and Usdin, 2010).

Models for the initiation of gene silencing fall into two basic groups, one in which the DNA itself recruits factors that ultimately result in the accumulation of repressive chromatin and the other in which RNA produced locally or distally in the form of short or long coding or non-coding RNAs is the trigger.

#### DNA-based models for the nucleation of silencing

**FIGURE 2:**
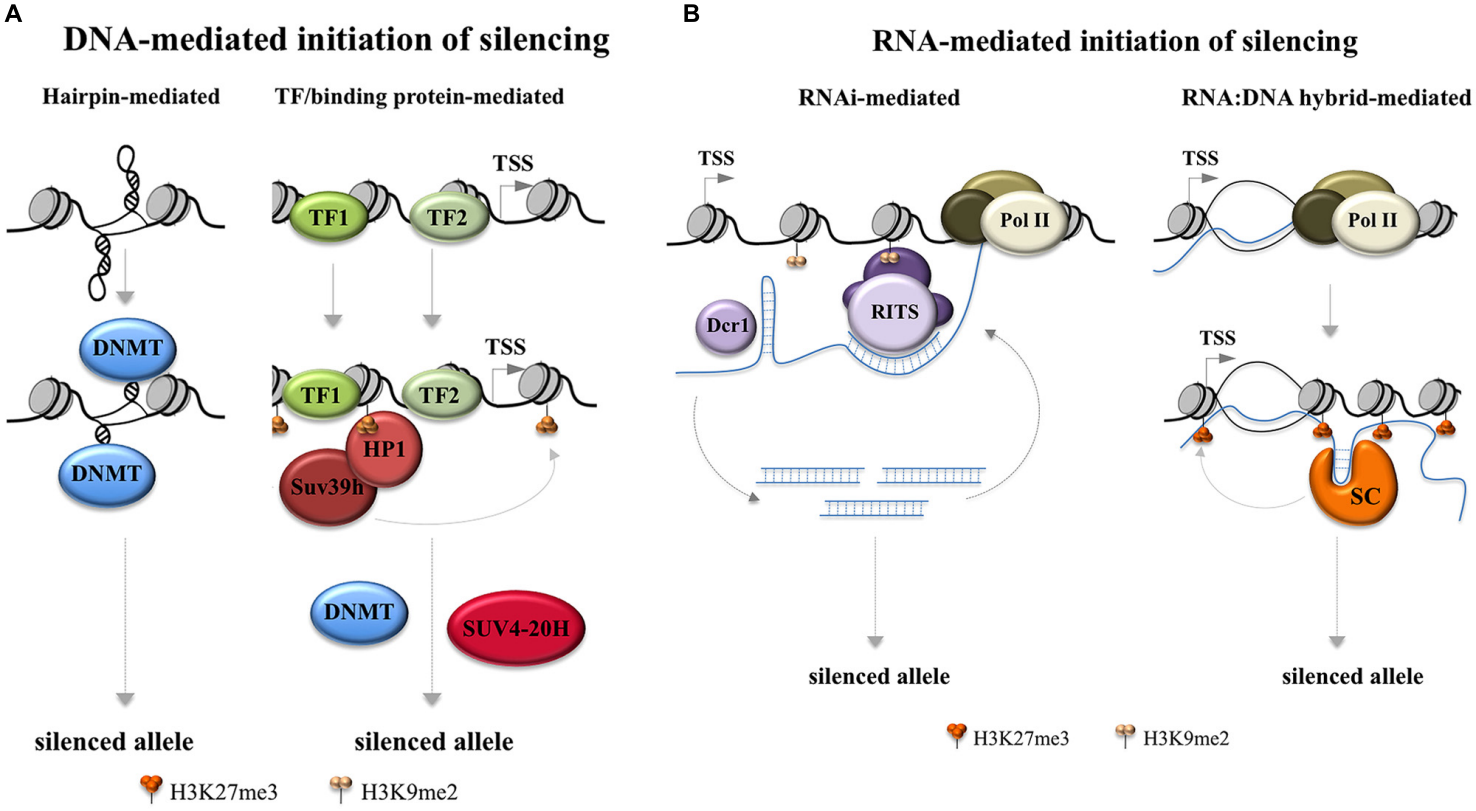
**Models for FX gene silencing.** Potential early steps in the initiation of FX gene silencing are depicted. **(A)** Two ways in which silencing can be triggered by the FX DNA. The left hand panel depicts a model based on the propensity of DNA methyltransferases to methylate FX hairpins *in vitro* (Smith et al., 1994; Chen et al., 1995). The right hand panel depicts a silencing scheme in which repeat binding proteins act to recruit silencing complexes based on the silencing mechanism that is thought to be responsible for the silencing of the major satellite repeats in pericentric heterochromatin in mice (Bulut-Karslioglu et al., 2012). In the case of the major satellite repeats recruitment of Suv39h leads to the recruitment of DNA methylases and SUV4-20H which trimethylates H4K20. DNMT: DNA methyltransferase; TF: transcription factor; TSS: transcription start site. **(B)** Two ways in which RNA-mediated gene silencing might occur in the FX locus. The left hand panel depicts an RNA interference based mechanism for gene silencing based on the model of silencing of centromeric tandem repeats in the fission yeast, Schizosaccharomyces pombe (Volpe et al., 2002). In the FX locus the RNA hairpins formed by the FX repeats in the *FMR1* transcript may be the source of the double-stranded Dicer (Dcr) substrates (Handa et al., 2003) as illustrated. In this case the annealing of the small Dicer products to the *FMR1* mRNA would occur via the same combination of Hoogsteen and Watson–Crick base pairing that generates the RNA hairpins in the first place. Alternatively, the duplex RNAs could be generated by base pairing of *FMR1* mRNA with an antisense transcript generated from this region (Ladd et al., 2007). The RNA-induced transcriptional silencing (RITS) complex, which includes the argonaute family member AGO1, then could mediate heterochromatin formation by associating with nascent transcripts via base pairing with the Dcr products. AGO1 could then recruit other epigenetic modifiers including members of the Polycomb (PcG) Group Complexes including EZH2 as suggested by work in human cells (Kim et al., 2006). The right hand panel depicts a way in which an RNA:DNA hybrid may initiate silencing by tethering the *FMR1* transcript to the *FMR1* locus while also recruiting silencing complexes (SC) that bind to the repeat, perhaps to the secondary structures formed by the repeat, analogous to what has been reported for the RASSF1A locus (Beckedorff et al., 2013) and for ribosomal DNA repeats and IAP elements (Bierhoff et al., 2014). Dcr1: Dicer 1; Pol II: RNA Polymerase II; RITS: RNA interference (RNAi) effector complex; SC: silencing complex; TSS: transcription start site.

The secondary structures formed by the repeats are known to be particularly good substrates for DNA methyltransferases *in vitro* (Smith et al., 1994). This has led to a model in which hairpin formation by the repeats in DNA triggers *de novo* DNA methylation as the first step in the silencing process as illustrated in the left hand panel of **Figure 2A**. However, if DNA methylation were in fact a later event in the silencing process, then this model would presumably not apply.

An alternate model for DNA-based initiation of silencing is seen in pericentromeric repeats in mice that occurs when the transcription factors Pax3 and Pax9 bind to the repeats and recruit the H3K9 trimethylase, Suv39h1 (Bulut-Karslioglu et al., 2012) as illustrated in the right hand panel of **Figure 2A**. The repeats that bind Pax3 and Pax9 are A+T-rich making it unlikely that the same factors bind to the FX repeats. However, it has been suggested that other repeat-binding proteins may act in the same way to bring about gene silencing (Bulut-Karslioglu et al., 2012).

#### RNA-based models for the nucleation of silencing

The *FMR1* locus produces a complex mixture of sense and antisense transcripts that could potentially trigger gene silencing in a variety of other ways (Ladd et al., 2007; Kumari and Usdin, 2010; Pastori et al., 2014). The CGG-repeats in RNA can form hairpins that are substrates for the enzyme Dicer, an important component of the RNA interference (RNAi) pathway (Handa et al., 2003). In mammals this pathway is usually associated with the post-transcriptional regulation of mRNAs but in fission yeast it can also lead to transcriptional gene silencing of centromeric repeats (Volpe et al., 2002). Dicer processes RNAs with double-stranded character into small interfering RNAs that, at least in yeast, are loaded onto the RNA-induced transcriptional silencing (RITS) complex. This complex can lead to gene silencing via the recruitment of Swi6/HP1 and a protein with similarities to the mammalian Suv39h protein (Nakayama et al., 2001; Rougemaille et al., 2012). Whether such a system operates in mammals is the subject of some debate. Work in human cells suggests that an analogous process does operate as illustrated in the right hand panel of **Figure 2B** (Kim et al., 2006). However, no evidence of abnormal methylation is seen at other CGG-repeat tracts in the human genome (Alisch et al., 2013). It is possible that the repeat number at any given locus has to exceed a certain threshold in order to be susceptible to silencing (Alisch et al., 2013). Such a threshold effect might also explain why the FM alleles are not silenced in mouse models for expanded repeats (Entezam et al., 2007; Brouwer et al., 2008). It is also possible that the silencing mechanism acts only *in cis*. Work done in neurons differentiated from FX ESCs suggested that Dicer is not required for either shutting down transcription of the *FMR1* gene or for dimethylation of H3K9 (Colak et al., 2014). However, since these cells were already at least partially methylated before differentiation began, a role for Dicer in the earliest steps of the silencing process cannot be excluded.

A number of Dicer-independent RNA-mediated silencing mechanisms are also possible. One such mechanism is related to the reported ability of the CGG-repeats to form persistent RNA:DNA hybrids (R-loops; Colak et al., 2014; Groh et al., 2014; Loomis et al., 2014). An R-loop containing the repeat is seen at the *FMR1* locus in neurons derived from FX ESCs. This R-loop appears after 45 days of differentiation and is followed by silencing of the *FMR1* gene (Colak et al., 2014). In these experiments no R-loop was seen in normal ESCs or cells from patients with FXTAS. A small molecule that is thought to block R-loop formation prevents gene silencing supporting the idea that the R-loop is crucial for this process. However, since some DNA methylation is already apparent in the ESCs prior to differentiation, it is likely that other events in the silencing process precede the action of the RNA:DNA hybrid. Why the R-loop is only seen in this small window of time is unclear, particularly since they can be readily detected in differentiated dermal fibroblasts even on shorter alleles (Loomis et al., 2014). Furthermore, why so much time elapses between the initiation of DNA methylation and the other epigenetic modifications that lead to silencing is also perplexing. However, there are a number of ways that R-loops could trigger silencing of the FX allele. They may cause transcription termination directly as do the R-loops generated by the repeat that causes the Repeat Expansion Diseases, amyotrophic lateral sclerosis (ALS) and frontotemporal lobar degeneration (FLTD; Haeusler et al., 2014) or recruit a transcriptional repressor as suggested for the COOLAIR locus in Arabidopsis (Sun et al., 2013). Reduced transcription could then result in the accumulation of repressive histone marks. It is also possible that silencing occurs due to the ability of RNA:DNA hybrids to recruit H3S10P, a mark of chromatin compaction (Castellano-Pozo et al., 2013).

An alternative model for FX gene silencing is suggested by what is known about silencing of the RASSF1A gene in humans. This is accomplished by the formation of RNA:DNA hybrid just downstream of the start of transcription by transcription from an antisense promoter (Beckedorff et al., 2013). The tethered RNA binds and recruits Polycomb repressive complex 2 (PRC2) to the RASSF1A promoter resulting in gene silencing. An example of how an RNA:DNA hybrid could operate to recruit factors that initiate silencing at the *FMR1* locus is illustrated in the right hand panel of **Figure 2B**. The *FMR1* 5′ end has some of the hallmarks of typical PRC2 target genes, including a high G+C-content and a high density of CpG residues (Mendenhall et al., 2010). Furthermore, there is evidence to suggest that many RNAs that bind to PRC2 and lead to gene silencing *in cis* form stem-loop structures (Kanhere et al., 2010) that are reminiscent of those formed by CGG-RNA (Handa et al., 2003). Other candidate epigenetic modifiers that bind structured RNAs could act in much the same way, including Suv4-20h2, the methylase responsible for H4K20 trimethylation. Recruitment of this histone methyltransferase by a long non-coding RNA (lncRNA) is thought to be the trigger for silencing of rDNA repeats and intracisternal A-particle (IAP) elements (Bierhoff et al., 2014).

Whatever the gene silencing mechanism, evidence suggests that the extent of this process can be very variable. Some FM carriers do not complete the gene silencing process, showing evidence of H3K9 dimethylation, but no DNA methylation (Tabolacci et al., 2008a). These individuals tend to be unaffected or less affected than those having alleles that are fully silenced. Other individuals show methylation mosaicism in which some alleles are more heavily methylated than others (McConkie-Rosell et al., 1993; Nolin et al., 1994; Rousseau et al., 1994; de Vries et al., 1996; Tassone et al., 1999; Pretto et al., 2014). Since unmethylated alleles often still make significant amounts of FMRP (Tassone et al., 2000a; Tabolacci et al., 2008a), methylation mosaicism likely contributes to variation in the levels of FMRP expressed in patient cells that is potentially a source of variability in the clinical presentation.

A number of epigenetic modifying drugs with the potential to reactivate the *FMR1* gene are in clinical trials for the treatment of life-threatening diseases like cancer, as well as other repeat expansion diseases with a high early mortality like FRDA and spinal muscular atrophy (Hahnen et al., 2006; Deutsch et al., 2008). Phase I clinical trials of one such compound, the HDAC inhibitor RG2833, showed that it could reduce epigenetic gene repression in FRDA patients. It is likely that many of these epigenetic modifiers will be too risky to use in a disorder like FXS that is not life threatening. However, there are others in the pipeline that may be both therapeutically useful and relatively non-toxic. One such compound, nicotinamide (Vitamin B3), an inhibitor of SIRT1, an enzyme we have shown to be important in FX gene silencing (Biacsi et al., 2008), is currently in clinical trials for the treatment of FRDA (http://www.clinicaltrials.gov/ct2/show/NCT01589809). The development of better inhibitors of SIRT1 as well as other epigenetic modifying enzymes in the silencing pathway may one day provide a therapeutic option for the treatment of FXS. Recently a small molecule that stabilizes the structures formed by FX repeats in the *FMR1* transcript and thus reduces R-loop formation, has been shown to block gene silencing (Colak et al., 2014). While this molecule was unable to reactivate a silenced allele, it is possible that in combination with other epigenetic modifiers it may ultimately have therapeutic potential. Of course, any benefit to be gained by reactivation of the FX allele would have to be offset by the risk posed by expression of *FMR1* mRNA with long CGG-repeat tracts.

## CHROMOSOME FRAGILITY

Fragile sites are constrictions, gaps or breaks that are seen in metaphase chromosomes [see (Lukusa and Fryns, 2008) for a comprehensive review]. Many such sites are present in the human genome where they are frequently associated with chromosome breakpoints and translocations. Fragile sites are said to be common or rare based on their incidence in the population, with common fragile sites being ubiquitously present and rare fragile sites being confined to a much smaller subset of individuals. Fragile sites are usually classified in terms of which agents most effectively induce their expression. These agents include folate, aphidicolin, distamycin, and bromodeoxyuridine. The presence of a folate-sensitive fragile site on the long arm of the X chromosome in individuals with FXS was noted many decades before the *FMR1* gene was identified (Howard-Peebles and Stoddard, 1979; Sutherland and Ashforth, 1979; Turner et al., 1980). The fragile site can be seen in as many as 20% of cells depending on repeat number and growth conditions. This indicates that the underlying event is likely to be extremely frequent.

There is reason to think that the high incidence of TS seen in FM females (Dobkin et al., 2009) could be a direct consequence of the chromosome fragility. For example, simultaneous breaks on both chromatids can lead to chromatid fusion, and the generation of a dicentric chromosome. The two centromeres of the chromosome will try to migrate toward opposite poles at anaphase. Chromosome aneuploidy can then occur through non-disjunction, if the dicentric chromatid is only released from one pole, resulting in one daughter cell receiving the whole chromosome at the expense of the other daughter cell. Alternatively, if the dicentric chromatid frees itself from the microtubules of both spindle poles, it can be lost from both cells as a result of anaphase lagging.

There are some clues as to what causes the FX allele to become fragile and/or break. Folate is a critical precursor for the synthesis of thymidine and too much or too little of this vitamin can cause nucleotide pool imbalances (James et al., 1992) that might cause or exacerbate problems with replication (Chabosseau et al., 2011) through the repeat. The FX repeats cause replication fork stalling in yeast and human cells (Voineagu et al., 2009). They also block initiation of replication from a major ORI located just upstream of the repeat in the *FMR1* gene itself and likely affect replication fork progression as well (Yudkin et al., 2014). This block is seen even in cells grown in the absence of folate-stress and thus the replication problem is likely an intrinsic feature of the repeats that occurs frequently under normal growth conditions. Folate-stress could induce fragile site expression by making an already late-replicating region (Webb, 1992; Hansen et al., 1993) replicate even later. This would increase the fraction of cells that enter mitosis before replication of this region is complete. What is responsible for the replication defect is unknown. R-loops, such as those formed by the FX repeats (Groh et al., 2014; Loomis et al., 2014), can stall DNA replication (Gan et al., 2011). However, chromosome fragility is more frequently seen on methylated FM alleles than unmethylated ones (Yudkin et al., 2014). It is unclear whether the residual transcription from methylated alleles is sufficient to cause stalling at a significant frequency. It is also possible that the secondary structures that can be formed by the FX repeat are responsible for the replication problem since they do form very stable blocks to DNA polymerases *in vitro* (Usdin and Woodford, 1995). Whatever their molecular basis, stalled replication forks can result in double-strand breaks generated in an attempt to rescue the fork. Such breaks could then lead to sister chromatid fusion and ultimately the loss of the X chromosome from a fraction of the daughter cells.

Thus each cell division poses a risk for the loss of the chromosome carrying the FM. If chromosome loss happens in the very early embryo, it is unlikely to survive (Hook and Warburton, 1983). However, if it occurs later in development, females will be mosaic for the loss of the X chromosome. The extent to which she would experience the symptoms of TS would depend on the fraction of her cells that are 45, X0.

## CONCLUDING REMARKS

As evidenced above, the presence of CGG/CGG -repeats at the 5′ end of the *FMR1* gene causes a number of genetic and epigenetic changes that can have profound effects on the *FMR1* locus and *FMR1* expression. These effects are, for the most part, expressed in direct proportion to the number of repeats in the affected allele. Thus unlike point mutations or insertions/deletions, the dynamic nature of the expansion mutation essentially makes the FX repeat a continuous variable, at least on the population level. Thus perhaps it should not be surprising that the symptoms seen in carriers of larger than normal numbers of CGG/CCG repeats form a continuum. Therefore, it may be more useful to think of as these individuals being somewhere on the Fragile X spectrum, such that the symptoms experienced may range from a risk of neurodegenerative changes or diminished ovarian function at one end of the spectrum to severe neurodevelopmental problems at the other. The range of symptoms displayed would depend on the number of repeats in the affected allele and the extent of somatic mosaicism that would impact the amount of *FMR1* mRNA and FMRP produced in critical cell types. A better understanding of all of the biological consequences of the repeats at both the DNA and RNA level may help us understand which of the effects can be mitigated and which ones cannot.

## Conflict of Interest Statement

The authors declare that the research was conducted in the absence of any commercial or financial relationships that could be construed as a potential conflict of interest.
